# Luteolin Effects on Mortality, Development and Population Parameters of *Frankliniella occidentalis* (Pergande)

**DOI:** 10.3390/insects16121255

**Published:** 2025-12-11

**Authors:** Xiaoyun Ren, Min Li, Li Zheng, Jincheng Zhou, Shengyong Wu, Xinbao Wei, Xunbing Huang, Haitao Yu

**Affiliations:** 1 College of Agriculture and Forestry Science, Linyi University, Linyi 276000, China; renxiaoyunyouxiang@163.com (X.R.); 13616491198@163.com (M.L.); zhengli64@126.com (L.Z.); parasitoidswasp@163.com (J.Z.); 2 State Key Laboratory for Biology of Plant Diseases and Insect Pests, Institute of Plant Protection, Chinese Academy of Agricultural Sciences, Beijing 100193, China; sywu@ippcaas.cn; 3 National Hainan Research Institute (Sanya), Chinese Academy of Agricultural Sciences, Sanya 572024, China; 4 Gansu Fengmiao Ecological Agriculture Development Co., Ltd., Lanzhou 730070, China; weixinbao_bx@163.com; 5 Institute of Plant Protection, Gansu Academy of Agricultural Sciences, Lanzhou 730070, China

**Keywords:** thrips, flavonoid, botanical pesticide, inhibiting population growth

## Abstract

Luteolin, as one of the flavonoids, has demonstrated repellent and toxic effects on various insect pests; however, its influence on thrips development and fecundity remains poorly understood. In this study, we investigated the toxicity of luteolin on the survival of a worldwide insect pest, the western flower thrips (WFT) (*Frankliniella occidentalis* Pergande), its effects on WFT feeding and oviposition preferences, and its sublethal impacts on development and fecundity. Results showed that both adult and nymphal WFT mortality rates increased with luteolin concentration. In choice assays, luteolin exhibited repellent effects on nymphal feeding and adult oviposition. At a sublethal concentration of 0.1 mg/mL, luteolin significantly shortened the adult lifespan, and reduced fecundity, intrinsic rate of increase (*r*), finite rate of increase (*λ*), and net reproductive rate (*R*_0_), suppressing WFT population growth. This study demonstrates that luteolin has the potential to be developed as a botanical pesticide for thrips management, combining with toxic and reproduction-inhibiting activities.

## Simple Summary

Flavonoids have demonstrated repellent and toxic effects on various insect pests; however, their influence on thrips remains poorly understood. Luteolin, as one of the flavonoids, has been reported to exhibit antifeedant activity and suppress insect pest population growth. In this study, we investigated the toxicity of luteolin on the survival and behavior of *Frankliniella occidentalis* (western flower thrips, WFT), as well as its effects on their development and fecundity. The results indicate that luteolin exhibits insecticidal activity against thrips. Furthermore, at a sublethal concentration (0.1 mg/mL), it adversely affects key life history traits, including adult longevity and fecundity, suppressing population growth. This study highlights the potential of luteolin as a botanical pesticide for thrips management.

## 1. Introduction

*Frankliniella occidentalis* (Pergande) (western flower thrips, WFT) is a globally distributed polyphagous pest. WFT damages plants through feeding and oviposition, causing silvered or necrotic patches on plant tissues, which reduces plant yield and quality [1,2]. WFT can transmit 11 plant viruses, including tomato chlorotic spot orthotospovirus (TCSV), tomato spotted wilt orthotospovirus (TSWV), tomato yellow ring virus (TYRV), etc. [3]. The biological characteristics—cryptic feeding, soil-dwelling pupal stage, small size, and high reproductive rate—make this pest particularly challenging to control [1,4,5]. Pesticide application is a common approach to control thrips, and improper practices (e.g., frequent spraying or overuse) have led to a high level of resistance in thrips [6]. Consequently, there is growing interest in developing innovative control strategies that are both environmentally sustainable and highly efficient [4,7].

Plant-derived components have been proposed for application in plant protection [8]. When subjected to biotic and abiotic stresses, plants employ a range of defense strategies, such as modulating secondary metabolism through the up- or down-regulation of metabolic pathways to release allelochemicals [8,9,10]. These chemicals contribute directly or indirectly to plant self-protection [9,11,12]. The metabolites, including the phenolics, terpenoids, and alkaloids, demonstrated pesticidal, repellent, and antifeedant activities, as well as the ability to inhibit insect population growth [13,14,15]. Flavonoids, a class of phenolic compounds, are nutritional compounds and pigments and are also involved in plant-insect pest interactions [16]. Certain flavonoids have been shown to confer resistance against insect pests. For example, high concentrations of naringenin and quercetin reduce fecundity and impair development in *Acyrthosiphon pisum* (Harris) [17]. Similarly, phloridzin lowers both fecundity and survival in *A. pisum* at high concentrations [18]. Furthermore, apigenin exerts an antifeedant effect on *Myzus persicae* (Sulzer) [19], while genistein disrupts the feeding behavior of the pea aphid *A. pisum*, also exhibiting a concentration-dependent antifeedant effect [20].

As a polyphenolic flavonoid, luteolin exhibits diverse biological activities, including antibacterial, anti-inflammatory, and anticancer effects [21,22]. Additionally, luteolin acts as an antifeedant against insect pests and suppresses pest population growth [23]. In vitro assays have demonstrated that luteolin prolongs the stylet probing period and reduces salivation in aphids such as *M. persicae* [19], *A. pisum* [20], and *Aphis fabae* (Scopoli) [24], exhibiting repellent feeding activity. Luteolin can also deter aphid settling and impede development in *Aphis gossypii* [23].

Notably, a high luteolin content has been identified in the resistant cowpea variety (IZJU0044), and its biosynthesis can be further stimulated by thrips damage [25]. In vitro assays have confirmed the toxicity of luteolin against *Megalurothrips usitatus* (Bagnall), and luteolin has been shown to be an important antifeedant compound against this thrips species [25], highlighting its potential as an eco-friendly control agent. However, the prospective use of luteolin as a botanical pesticide against thrips requires a comprehensive study. Therefore, this study investigated the bioactivity of luteolin against WFT. Through no-choice and choice assays, the effects of luteolin on feeding and oviposition preference were examined. Furthermore, the impact of luteolin on WFT development and fecundity was evaluated using a life table study [26,27].

## 2. Materials and Methods

### 2.1. Insect Rearing

WFT were collected from *Capsicum annuum* L. in Beijing and reared with fresh bean (*Phaseolus vulgaris* L.) pods in Linyi University (35°7′21″ N, 118°17′15″ E) in Shandong Province, China. WFT were kept in a climate chamber set at 25 ± 1 °C, 65% relative humidity (RH), and a photoperiod of 14:10 h (L:D).

### 2.2. Bioassay of the Effect of Luteolin on WFT

**Figure 1:**
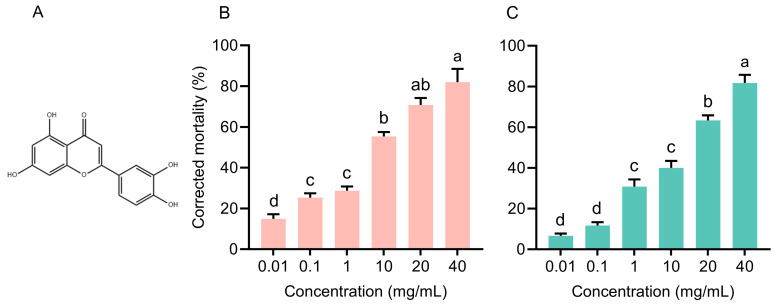
Chemical structure (**A**) and bioassay of luteolin on *Frankliniella occidentalis* nymphs (**B**) and adults (**C**). Data are presented as the mean ± SE. Different lower cases indicate concentration caused different effects on *F. occidentalis* mortality (*p* < 0.05).

A series of luteolin (Figure 1A) (Shanghai Macklin Biochemical Co., Ltd., Shanghai, China) solutions (40, 20, 10, 1, 0.1, and 0.01 mg/mL) were prepared using 75% ethanol (Sinopharm Chemical Reagent Co., Ltd., Shanghai, China) to evaluate the biopesticide effect of luteolin on WFT. Bean pods were cut into 1 cm × 0.5 cm sections (without seed), dipped into the luteolin solvents for 10 s, and air-dried under laboratory conditions. The 5 mL centrifuge tubes were coated with the luteolin solutions and dried under the same conditions [25]. Twenty WFT adults or 1st instar nymphs were transferred into each tube along with a treated bean pod section. The tube lid was covered by a fine transparent mesh screen to ensure adequate ventilation. Mortality was assessed after 48 h by counting the number of dead and surviving thrips. The control group was exposed to bean pods and centrifuge tubes treated only with 75% ethanol (0 mg/mL luteolin). Each concentration was tested with six replicates for both nymphs and adults.

Based on the results from this section, 0.01 luteolin at 0.1 mg/mL caused corrected mortality rates of 14.95–25.26% and 6.67–11.67% in nymphs and adults, respectively. These two concentrations were selected to investigate the sublethal effects of luteolin on WFT.

### 2.3. Luteolin on WFT Feeding and Oviposition Selection

Both no-choice and choice assays were used to determine whether luteolin can repel WFT. A 2.5 mL volume of 1.2% water agar (Beijing Solarbio Science & Technology Co., Ltd., Beijing, China) was poured into Petri dishes (9 cm diameter) to maintain humidity [28] and prevent leaf curling. In no-choice assays, *P. vulgaris* leaf disks with a diameter of 1 cm were dipped into luteolin solutions (0, 0.01, and 0.1 mg/mL). After drying, a single disk was placed in a Petri dish. Ten starved WFT nymphs or five pairs of adults were then released into each dish. The lids were sealed with fine mesh for ventilation and Parafilm around the edges to prevent escape. After 24 h, the feeding damage area on the leaf disks caused by nymphs was measured under a stereo microscope (Leica EZ4, Leica Microsystems, Wetzlar, Germany). For adults, the WFT were removed from the leaf disks after 24 h. The leaf disks were then observed daily, and the number of hatched nymphs was recorded until no new nymphs emerged. The total number of nymphs was taken as the number of eggs laid by WFT adults.

In the dual-choice assay, two control leaf disks and two disks treated with either 0.01 or 0.1 mg/mL luteolin were placed opposite each other in a Petri dish. Ten WFT nymphs or adults were released at the center. After 24 h, the total feeding area (for nymphs) and the number of eggs laid (for adults) were assessed. Each Petri dish was considered as one replicate, and 10 replicates were performed for each treatment.

### 2.4. Sublethal Effect of Luteolin on WFT

Approximately 100 adult WFT were transferred to bean pods treated with 0, 0.01, or 0.1 mg/mL luteolin. After a 24 h oviposition period, the adults were removed. Newly hatched nymphs from each treatment group were individually transferred to separate Petri dishes (6 cm in diameter) and monitored at 24 h intervals. Survival and development of the thrips were recorded daily, and the treated bean pods were refreshed accordingly. Upon adult emergence, the sex of WFT was determined, and individuals were paired according to the methodology of Ren et al. (2025) [29]. The longevity and daily fecundity of adult WFT in each treatment were recorded until death. For the life table study, each treatment group initially consisted of 40 newly hatched nymphs.

### 2.5. Data Analysis

Data analysis, including corrected mortality, damage area, and damage choice, was performed using SPSS v.17.0 (SPSS Inc., Chicago, IL, USA). Corrected mortality was calculated using Abbott’s formula [30]. The normality of all data was checked with the Shapiro–Wilk test. For the bioassay data (corrected mortality) and no-choice assay data (including feeding damage area and the number of eggs oviposited), one-way analysis of variance (ANOVA) followed by Tukey’s HSD post hoc test was employed to assess significant differences among luteolin concentrations at a significance level of *p* < 0.05. Data from the dual-choice assays were analyzed using a paired *t*-test (*p* < 0.05). The effects of luteolin on WFT development, reproduction, and population parameters were evaluated using the TWOSEX-MSChart statistical software (https://www.faas.cn/cms/sitemanage/index.shtml?siteId=810640925913080000, accessed on 18 February 2025) [31,32]. The intrinsic rate of increase (*r*) [33], net reproduction rate (*R*_0_), finite rate of increase (*λ*), and mean generation time (*T*) were calculated using the following formulas:

(1) $$\sum_{x=0}^{\infty }e^{-r(x+1)}l_{x}m_{x}=1$$

*r* is estimated iteratively according to the Euler–Lotka equation with an age index from 0 [22], and this formula is used to calculate *r*.


(2) $$\lambda =e^{r}$$

(3) $$R_{0}=\sum_{x=0}^{\infty }l_{x}m_{x}$$

*R*_0_ is the average number of offspring produced by one insect during its lifetime.

(4) $$T=\frac{ln(R_{0})}{r}$$

*T* is the time a population needs to increase to *R*_0_ times.

The paired bootstrap test was used to determine the differences between treatments.

## 3. Results

### 3.1. Effect of Luteolin on WFT Mortality

The corrected mortality of both nymphs and adults increased with increasing luteolin concentration (Figure 1B,C). For nymphs (Figure 1B), corrected mortality did not differ significantly between the 0.1 and 1 mg/mL luteolin treatments, but both were significantly higher than that at 0.01 mg/mL. For adults, the corrected mortality at 1 mg/mL was significantly higher than that at 0.1 mg/mL (Figure 1C). At a concentration of 40 mg/mL, luteolin caused over 80% corrected mortality in both nymphs and adults. The LC50 values of luteolin to WFT nymphs and adults were 2.062 mg/mL and 5.678 mg/mL, respectively (Table 1).

### 3.2. Luteolin Effect on WFT Feeding Damage

**Figure 2:**
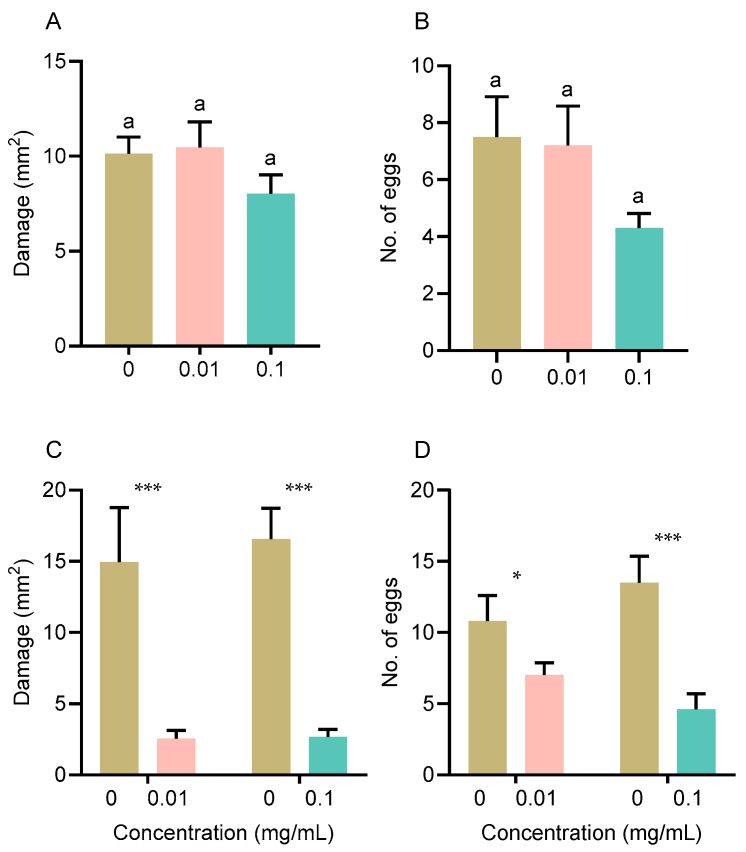
Luteolin effect on feeding area damaged by nymphs ((**A**): no choice, (**C**): with choice) and No. of eggs laid by an adult in 24 h ((**B**): no choice, (**D**): with choice). Data are presented as the mean ± SE. Different lowercase letters (**A**,**B**) indicate statistically significant differences (*p* < 0.05), and asterisks (**C**,**D**) indicate significant differences between treatments (*** *p* < 0.001, * *p* < 0.05).

In the no-choice assay, no significant differences were observed in either nymphal feeding damage (Figure 2A: *F*_2,27_ = 1.491, *p* = 0.243) or adult oviposition (Figure 2B: *F*_2,27_ = 2.230, *p* = 0.127) across the different luteolin concentrations. Conversely, in the choice assay, WFT nymphs exhibited a significant feeding preference for the control leaf disks over those treated with luteolin (0.01 mg/mL: *t* = −12.410, *df* = 9, *p* = 0.013; 0.1 mg/mL: *t* = −6.023, *df* = 9, *p* < 0.001) (Figure 2C). Adults laid significantly more eggs on control leaf disks than on luteolin-treated disks (0.01 mg/mL: *t* = −2.467, *df* = 9, *p* = 0.036; 0.1 mg/mL: *t* = −5.309, *df* = 9, *p* < 0.001) (Figure 2D).

### 3.3. Luteolin Effect on the Developmental and Survival of WFT

The effects of luteolin on the developmental duration of WFT are presented in Table 2, and the corresponding coefficient of variation values are provided in the Supplementary File. No significant differences were observed in preadult developmental time (including egg, nymphal, pupal, and total preadult stages) or female adult longevity between the luteolin-treated and control groups. However, luteolin application significantly reduced the preadult survival rate of WFT. At 0.1 mg/mL, luteolin significantly shortened the adult duration, male longevity, and mean longevity of WFT compared to the control. In contrast, adult duration, male longevity, and mean longevity were not significantly affected by luteolin at 0.01 mg/mL.

### 3.4. Luteolin Effect on the Population Parameters of WFT

The population parameters of WFT were significantly influenced by luteolin concentration (Table 3), with the corresponding coefficient of variation values provided in the Supplementary File. At 0.1 mg/mL, luteolin significantly prolonged the total pre-oviposition period (TPOP) (11.42 d) but shortened the oviposition days (*O_d_*) (11.17 d) in WFT, compared to both the control group (TPOP: 10.62 d; Od: 12.52 d) and the 0.01 mg/mL luteolin group (TPOP: 10.88 d; *O_d_*: 11.75 d). Furthermore, compared to the control, both concentrations of luteolin (0.01 and 0.1 mg/mL) reduced WFT fecundity (*F*), intrinsic rate of increase (*r*), finite rate of increase (*λ*), and net reproductive rate (*R*_0_) to varying degrees. However, the reduction was more pronounced at 0.1 mg/mL, with significantly lower values for fecundity (48.62 eggs/female), *r* (0.1691 d^−1^), λ (1.1842 d^−1^), and *R*_0_ (15.800 offspring/individual) compared to the control (*F*: 70.24 eggs/female; *r*: 0.2352 d^−1^; *λ*: 1.2651 d^−1^; *R*_0_: 36.95 offspring/individual). The mean generation time (*T*) was not significantly affected by luteolin at either concentration.

## 4. Discussion

Flavonoids play crucial roles in plant responses to abiotic and biotic stresses, protecting plants from damage [18,34]. The pesticidal activity has been studied on more than 280 different flavonoid compounds, such as rutin, quercetin, apigenin, etc., and has been demonstrated [35,36]. In this study, we demonstrated that luteolin exhibits potential as a botanical pesticide against WFT, showing deterrent and growth-inhibitory activities.

The production of luteolin is upregulated in plants upon insect damage [23,25,37], and this compound displays insecticidal effects against insect pests, such as *Mythimna separata* (Walker), *Plutella xylostella* [38], *Spodoptera exigua* (Hübner) larvae [39], and *M. usitatus* [23]. Consistent with He et al. (2025) [25], our study confirmed luteolin pesticidal activity against WFT, and the LC50 values for WFT nymphs and adults at 48 h were 2.062 mg/mL and 5.678 mg/mL, respectively. Notably, the susceptibility to luteolin varies considerably among insect species. For instance, while the LC50 for *M. usitatus* adults is 6.0861 mg/mL, LC50 values of luteolin and luteolin glycosides for the 4th-instar larvae of *P. xylostella* are 0.0047 and 0.0046 mg/mL, respectively [38]. In comparison, the LC50 of luteolin against thrips was significantly higher than that against lepidopteran insects [23,38]. This could be because, in the thrips study, luteolin was applied to the plant surface, resulting in a lower intake of luteolin by the thrips. Subsequent studies could incorporate luteolin into artificial diets to further explore the toxicity mechanism of luteolin against thrips. Moreover, when used in combination with pesticides, they can enhance pest control efficacy [40,41]. Therefore, research on the combined application of luteolin with other insecticides for the control of thrips can be conducted.

Highly resistant plant varieties often constitutively produce elevated levels of flavonoids, which can reduce insect preference and fitness [37,42]. Field studies have linked high polyphenol content in cowpea to resistance against *Megalurothrips sjostedti* (Trybom) [42], and a lower abundance of *M. usitatus* has been observed on resistant cowpea varieties with high flavonoid content [25]. Specific flavonoids, including phloridzin, phloretin, naringenin, catechin, genistein, quercetin, apigenin, and rutin, are known to deter insect feeding [19,20,23,43]. Luteolin and its glycosides exhibit antifeedant effects against aphids by prolonging the time to the first ingestion and the duration of active feeding [19,23,24]. Similarly, a luteolin glycoside from resistant sweet pepper showed oviposition repellency against *Lirimoyza trifolii* [44]. In our study, luteolin significantly deterred WFT in choice assays, reducing both nymphal feeding damage and adult egg deposition. Flavonoids can, however, under no-choice conditions, although the tested concentrations did not cause significant differences in nymphal feeding or adult oviposition, the higher concentration (0.1 mg/mL) showed a trend of reducing both parameters. For *Spodoptera frugiperda* (J.E. Smith), larvae consumed similar quantities of leaves treated with different quercetin concentrations under no-choice conditions, whereas significant feeding inhibition was observed only at high concentrations in choice assays [45]. When given a choice, the larvae strongly preferred untreated leaves or those treated with a low concentration of pinocembrin (0.1 μg/cm^2^). In contrast, higher concentrations of pinocembrin (5 and 50 μg/cm^2^) effectively inhibited their feeding preference [45]. These results demonstrate that the repellent efficacy of flavonoids depends on both the specific compound and its concentration.

Flavonoids are known to adversely affect the survival, development, and fecundity of insect pests [17,37]. Consistent with this, our life table analysis revealed that luteolin negatively influenced the development and population parameters of WFT. Although luteolin did not significantly alter preadult developmental durations, it caused high preadult mortality, indicating direct toxicity to WFT, which aligns with our laboratory bioassays and a previous study [25]. The absence of a significant effect on preadult development has also been observed in *A. gossypii* treated with luteolin and several other flavonoids [23], whereas luteolin was reported to inhibit larval growth and development in *S. exigua* (Hübner) [39]. Overall, while the prolongation of preadult duration was not statistically significant, luteolin notably shortened the adult duration and significantly reduced male longevity. At 0.1 mg/mL, luteolin prolonged the total pre-oviposition period (TPOP) to 11.42 days. This concentration also significantly decreased oviposition days (*O_d_*), fecundity, and key population parameters (*r*, *λ*, and *R*_0_), thereby delaying population growth. Similar suppression of population growth by luteolin has been reported in *A. gossypii* [23]. Likewise, the high-flavonoid resistant cowpea variety also inhibits population growth in *M. usitatus* [25]. The underlying mechanisms of flavonoids on insect pest behavior, growth, and development may involve the modulation of enzyme activity [46], inhibiting ecdysone receptor-dependent gene transcription [47], and antimicrobial activity [16,48], which should be investigated further.

## 5. Conclusions

This study confirms that luteolin possesses insecticidal activity against thrips and, at sublethal concentrations (0.1 mg/mL), adversely affects key life history traits—including adult longevity and fecundity—leading to the suppression of population increase. It is noteworthy that while significant behavioral deterrence was absent in no-choice assays, a trend of reduced feeding and oviposition was observed at higher concentrations. These results highlight that luteolin has the potential to be developed as a botanical pesticide for thrips management.

## Figures and Tables

**Table 1:** Toxicity of the luteolin to *Frankliniella occidentalis* nymphs and adults.

Thrips Stage	48 h-LC50 ^1^ (95% CI ^2^) (mg/mL)	*χ* ^2^	*p*	*df*	Slope (SE)	*R* ^2^
Nymphs	2.062 (1.300 ~ 3.343)	39.707	0.231	34	0.490 (0.041)	0.742
Adult	5.678 (3.835 ~ 8.964)	30.338	0.734	36	0.638 (0.049)	0.930

^1^ 48 h-LC50, 48 h median lethal rate. ^2^ 95% CI, 95% confidence interval.

**Table 2:** Effect of luteolin on the developmental times (d), longevities (d) and preadult survival rate (mean ± SE) of *Frankliniella occidentalis*.

Stage	Control		0.01 mg/mL		0.1 mg/mL	
	n	Mean ± SE	n	Mean ± SE	n	Mean ± SE
Egg	40	3.05 ± 0.03 a	40	3.15 ± 0.06 a	40	3.15 ± 0.06 a
Nymph	38	3.89 ± 0.21 a	25	3.67 ± 0.22 a	31	3.94 ± 0.26 a
Pupa	33	3.15 ± 0.05 a	23	3.17 ± 0.08 a	21	3.29 ± 0.14 a
Preadult	33	10.27 ± 0.25 a	23	10.48 ± 0.16 a	21	10.76 ± 0.23 a
Preadult survival (%)	40	82.5 ± 6.02 a	40	57.5 ± 7.79 b	40	52.5 ± 7.90 b
Adult	33	14.06 ± 0.87 a	23	12.74 ± 1.01 ab	21	11.00 ± 1.20 b
Male total longevity	12	22.75 ± 1.15 a	7	19.29 ± 1.51 b	8	18.75 ± 1.73 b
Female longevity	21	25.24 ± 1.23 a	16	24.94 ± 1.06 a	13	23.62 ± 1.36 a
Mean longevity	40	21.23 ± 1.32 a	40	15.95 ± 1.48 b	40	14.70 ± 1.35 b

Note: Different lowercase letters within the same row indicate significant differences between the two treatments (paired bootstrap test, *B* = 100 000, *p* < 0.05).

**Table 3:** Effect of luteolin on the population parameters (Mean ± SE) of *Frankliniella occidentalis*.

Parameters	Control		0.01 mg/mL		0.1 mg/mL	
	n	Mean ± SE	n	Mean ± SE	n	Mean ± SE
Total pre-oviposition period (TPOP) (d)	21	10.62 ± 0.30 a	16	10.88 ± 0.31 a	12	11.42 ± 0.23 b
Oviposition days (*O_d_*) (d)	21	12.52 ± 1.07 a	16	11.75 ± 1.01 a	12	11.17 ± 1.46 b
Fecundity (eggs/female)	21	70.24 ± 6.88 a	16	56.25 ± 4.72 ab	12	48.62 ± 7.95 b
Intrinsic rate of increase (*r*) (d^−1^)	40	0.2352 ± 0.0138 a	40	0.1963 ± 0.0151 ab	40	0.1691 ± 0.0185 b
Finite rate of increase (*λ*) (d^−1^)	40	1.2651 ± 0.0173 a	40	1.2170 ± 0.0182 ab	40	1.1842 ± 0.0218 b
Net reproductive rate (*R*_0_) (offspring/individual)	40	36.95 ± 6.5689 a	40	22.50 ± 4.7231 ab	40	15.800 ± 4.3576 b
Mean generation time (*T*) (d)	40	15.339 ± 0.5140 a	40	15.756 ± 0.441 a	40	16.321 ± 0.3170 a

Note: Different lowercase letters within the same row indicate significant differences between the two treatments (paired bootstrap test, *B* = 100 000, *p* < 0.05).

## Data Availability

The original contributions presented in this study are included in the article/Supplementary Materials. Further inquiries can be directed to the corresponding authors.

## Supplementary Materials

The following supporting information can be downloaded at https://www.mdpi.com/article/10.3390/insects16121255/s1, Table S1: Coefficient of variations (%) * of developmental times, longevities and preadult survival rate of *Frankliniella occidentalis*. Table S2: Coefficient of variations (%) * of population parameters of *Frankliniella occidentalis*.
