# Supplementary material for: Luteolin Effects on Mortality, Development and Population Parameters of Frankliniella occidentalis (Pergande)
Source: Insects. 2025 Dec 11;16(12):1255. doi: 10.3390/insects16121255 (PMC12734059; doi:10.3390/insects16121255)
Supplement: Supplementary file 1 [file insects-16-01255-s001.zip › insects-4032152-supplementary.pdf]

Supplementary file

Table S1. Coefficient of variations (%)* of developmental times, longevities and preadult survival rate of *Frankliniella occidentalis*

Stages	Control	0.01 mg/mL	0.1 mg/mL
Egg	6.22	12.05	12.05
Nymph	33.28	29.97	36.74
Pupa	9.12	12.10	19.50
Preadult	13.98	7.32	9.80
Preadult survival	46.15	85.68	95.17
Adult	35.55	38.02	49.99
Male total longevity	17.51	20.71	26.10
Female longevity	22.33	17.00	20.76
Mean longevity	39.32	58.69	58.08

* Coefficient of variation (CV) was calculated using the following formula: $CV(\%) = \frac{SD}{Mean} \times 100$. Additionally, the relationship between SE and SD is given by $SE = \frac{SD}{\sqrt{n}}$.

Table S2. Coefficient of variations (%)* of population parameters of *Frankliniella occidentalis*

Population parameters	Control	0.01 mg/mL	0.1 mg/mL
Total pre-oviposition period (TPOP)	12.94512908	11.39706	6.976737
Oviposition days (O_d)	39.16418525	34.38298	45.27832
Fecundity	44.88627674	33.56444	56.64255
Intrinsic rate of increase (r)	37.1083603	48.65043	69.19236
Finite rate of increase (λ)	8.648708169	9.45825	11.64291
Net reproductive rate (R_0)	112.4367292	112.4367	174.4296
Mean generation time (T)	21.1931771	17.70201	12.28408

* Coefficient of variation (CV) was calculated using the following formula: $CV(\%) = \frac{SD}{Mean} \times 100$. Additionally, the relationship between SE and SD is given by $SE = \frac{SD}{\sqrt{n}}$.
